# 2-[5-(2,3-Di­meth­oxy­naphthalen-1-yl)-4,5-di­hydro-1*H*-pyrazol-3-yl]-3-meth­oxy­phenol

**DOI:** 10.1107/S2414314623006685

**Published:** 2023-08-10

**Authors:** Jiha Sung

**Affiliations:** a Department of Applied Chemistry, Dongduk Women’s University, Seoul 136-714, Republic of Korea; University of Aberdeen, United Kingdom

**Keywords:** crystal structure, pyrazoline, N—H⋯N hydrogen bonds, inversion dimers

## Abstract

In the title compound, C_22_H_22_N_2_O_4_, the central pyrazoline ring exhibits a nearly planar structure (r.m.s. deviation = 0.025 Å) despite having two *sp* ^3^ carbon atoms. The pyrazoline ring subtends dihedral angles of 4.61 (1) and 87.31 (1)° with the pendant benzene ring and naphthalene ring system, respectively. The dihedral angle between the planes of the benzene ring and the naphthalene ring system is 89.76 (2)°. An intra­molecular O—H⋯N hydrogen bond forms an *S*(6) ring motif. In the crystal, inversion dimers formed by pairwise weak N—H⋯N hydrogen bonds generate *R* ^2^_2_(4) loops and the dimers are linked by pairwise C—H⋯O hydrogen bonds [which generate *R* ^2^_2_(8) loops] into [100] chains.

## Abstract 2

In the title compound, the central pyrazoline ring subtends dihedral angles of 4.61 (1) and 87.31 (1)° with the pendant benzene ring and naphthalene ring system, respectively. In the crystal, inversion dimers formed by pairwise weak N—H⋯N hydrogen bonds are linked by pairwise C—H⋯O hydrogen bonds into [100] chains.

## Graphical abstract

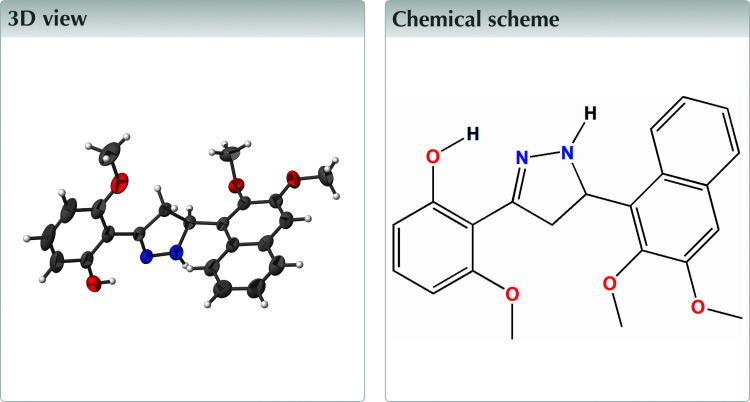

## Structure description

Pyrazolines have been reported to show a broad spectrum of biological activities including anti­cancer (Haider, *et al.*, 2022 ▸), anti­microbial (Bano *et al.*, 2015 ▸), anti-inflammatory (Viveka *et al.*, 2015 ▸), anti­malarial (Kumar *et al.*, 2018 ▸) and anti-Parkinsonian effects (Singh *et al.*, 2018 ▸). Pyrazoline is generally synthesized from chalcone, and various synthetic methods have been reported in the literature (Praceka *et al.*, 2021 ▸). Chalcones are key precursors for the synthesis of a various flavonoids when they have a hydroxyl group at the β-position of the ketone group. The single-crystal structures of various flavonoids synthesized from chalcones have previously been reported by our group (Sung, 2020 ▸). In a continuation of our research inter­est in broadening the application range of β-hydroxyl chalcone, the title pyrazoline compound was synthesized and its crystal structure was determined.

**Figure 1:**
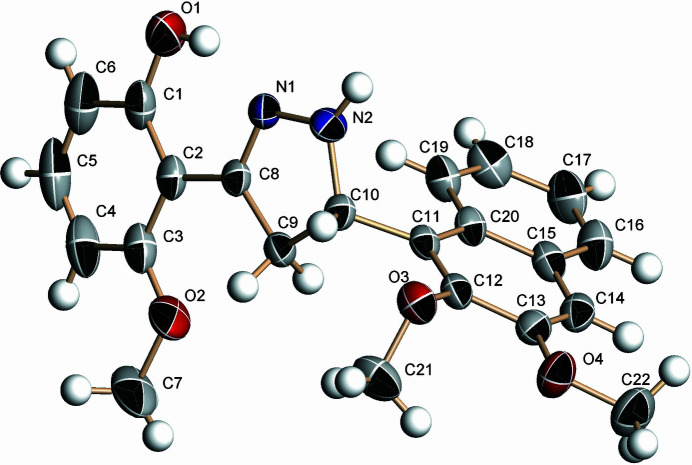
The mol­ecular structure of the title compound with displacement ellipsoids drawn at the 30% probability level.

**Figure 2:**
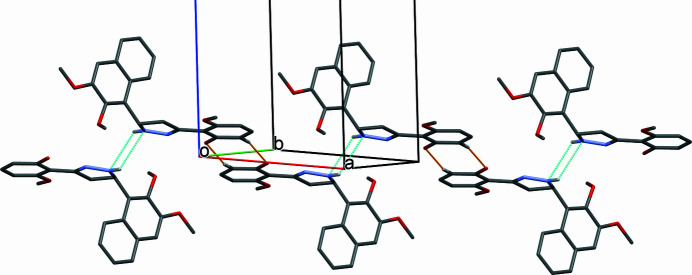
A partial view of the crystal structure of the title compound showing dimer chains of mol­ecules formed along [010]. Inter­molecular C—H⋯·O hydrogen bonds are shown as dashed lines (see Table 1 ▸).

The title mol­ecule, C_22_H_22_N_2_O_2_, crystallizes in space group *P*2_1_/*n* with one mol­ecule in the asymmetric unit (Fig. 1 ▸). The central pyrazoline ring contains two *sp* ^3^ carbon atoms (C9 and C10), but it has a nearly planar structure (r.m.s. deviation = 0.025 Å). The benzene ring and naphthalene ring system are attached at positions C8 and C10 of the pyrazoline ring, and they are tilted by 4.61 (1) and 87.31 (1)°, respectively, with respect to the mean plane of the pyrazoline ring. The dihedral angle between the planes of the benzene ring and naphthalene ring system is 89.76 (2)°. The meth­oxy groups at the 3-position of naphthalene ring and the *ortho* position of the benzene ring are almost coplanar with the rings to which they are bound [C—O—C—C = −7.9 (5) and −0.4 (4)°, respectively], whereas the meth­oxy group at the 2-position of the naphthalene ring system is twisted from the ring [C—O—C—C = 112.5 (3)°]. The hydroxyl group at the *ortho* position of the benzene ring makes an intra­molecular O1—H10⋯N1 hydrogen bond, forming an *S*(6) ring motif. In the crystal, inversion dimers linked by pairwise N2—H2*A*⋯N2 hydrogen bonds generate 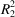 (4) loops and these dimers are linked by pairwise C6—H6⋯O1 hydrogen bonds [which generate 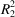 (8) loops] into [100] chains (Table 1 ▸, Fig. 2 ▸).

## Synthesis and crystallization

**Figure 3:**
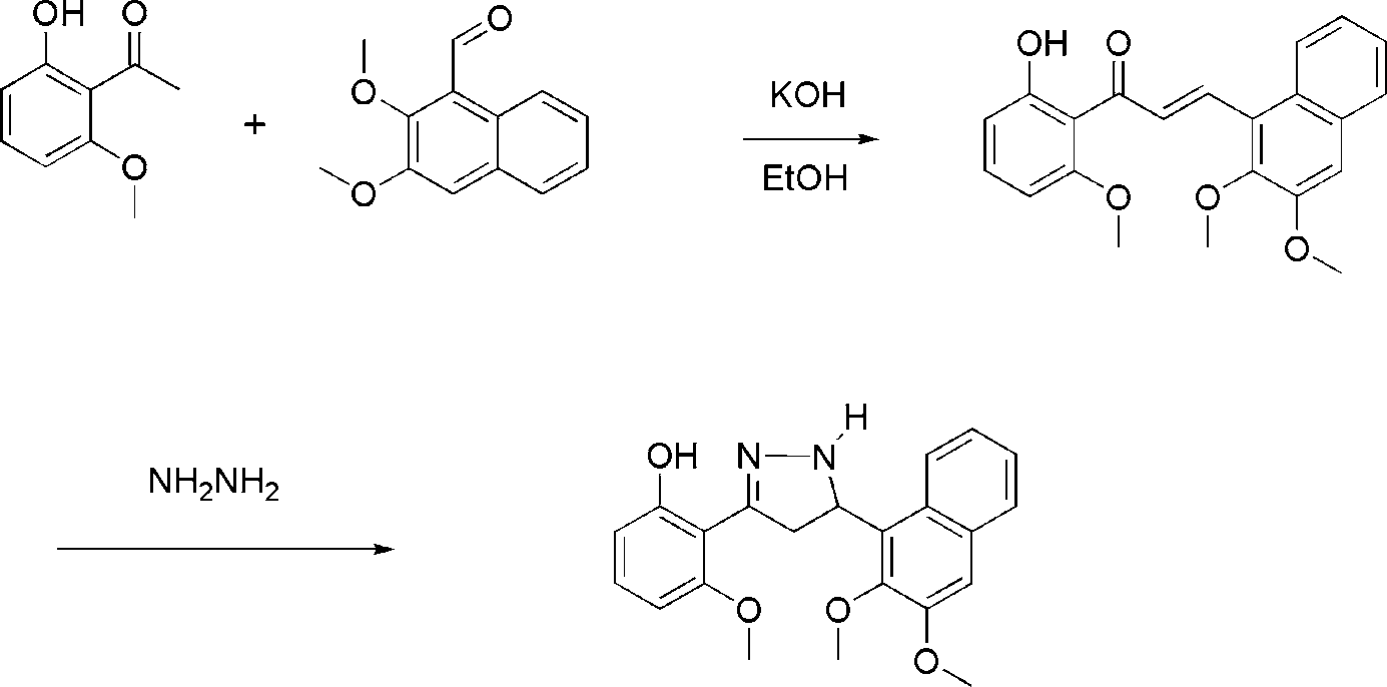
Synthetic scheme for preparation of the title pyrazoline compound.

The starting chalcone, (*E*)-3-(2,3-di­meth­oxy­naphthalen-1-yl)-1-(2-hy­droxy-6-meth­oxy­phen­yl)prop-2-en-1-one, was prep­ared by the previously reported method (Sung, 2019 ▸). Pyrazoline was synthesized by a cyclization reaction of the chalcone with NH_2_NH_2_ (Fig. 3 ▸). To a solution of 6-meth­oxy-2-hy­droxy­aceto­phenone (10 mmol, 1.66 g) in 50 ml of ethanol was added 2,3-dimeth­oxy-1-naphthaldehyde (10 mmol, 1.56 g) and the temperature was adjusted to around 276–277 K in an ice bath. To the reaction mixture were added 8 ml of 40% (*w*/*v*) aqueous KOH solution and reaction mixture was stirred at room temperature for 20 h. At the end of the reaction, ice water was added to the mixture and acidified with 6 *N* HCl (pH = 3–4). The resulting precipitate was filtered and washed with water and ethanol. The crude solid was purified by recrystallization from ethanol solution to give the pure chalcone. Excess hydrazine monohydrate (1 ml of 64–65% solution, 13 mmol) was added to a solution of the chalcone compound (5 mmol, 1.52 g) in 30 ml of anhydrous ethanol and the solution was refluxed at 360 K for 5 h. The reaction mixture was cooled to room temperature to yield a solid that was then filtered. The crude solids were purified by recrystallization from ethanol solution to afford the title compound.

## Refinement

Crystal data, data collection and structure refinement details are summarized in Table 2 ▸.

## Supplementary Material

Crystal structure: contains datablock(s) I. DOI: 10.1107/S2414314623006685/hb4442sup1.cif

Structure factors: contains datablock(s) I. DOI: 10.1107/S2414314623006685/hb4442Isup2.hkl

Supporting information file. DOI: 10.1107/S2414314623006685/hb4442Isup3.cml

CCDC reference: 2285981

Additional supporting information:  crystallographic information; 3D view; checkCIF report

## Figures and Tables

**Table 1:** Hydrogen-bond geometry (Å, °)

*D*—H⋯*A*	*D*—H	H⋯*A*	*D*⋯*A*	*D*—H⋯*A*
O1—H1⋯N1	0.84	1.81	2.556 (3)	148
N2—H2*A*⋯N2^i^	0.88	2.68	3.196 (5)	118
C6—H6⋯O1^ii^	0.95	2.50	3.433 (5)	166

Symmetry codes: (i) 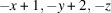 ; (ii) 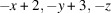 .

**Table 2:** Experimental details

Crystal data	
Chemical formula	C_22_H_22_N_2_O_4_
*M*_r_	378.42
Crystal system, space group	Monoclinic, *P*2_1_/*n*
Temperature (K)	200
*a*, *b*, *c* (Å)	9.6536 (9), 9.0435 (9), 21.599 (2)
β (°)	94.473 (2)
*V* (Å^3^)	1879.9 (3)
*Z*	4
Radiation type	Mo *K*α
μ (mm^−1^)	0.09
Crystal size (mm)	0.26 × 0.21 × 0.08

Data collection	
Diffractometer	Bruker SMART CCD
No. of measured, independent and observed [*I* > 2σ(*I*)] reflections	11235, 3687, 1933
*R*_int_	0.055
(sin θ/λ)_max_ (Å^−1^)	0.617

Refinement	
*R*[*F* ^2^ > 2σ(*F* ^2^)], *wR*(*F* ^2^), *S*	0.059, 0.184, 0.92
No. of reflections	3687
No. of parameters	257
H-atom treatment	H-atom parameters constrained
Δρ_max_, Δρ_min_ (e Å^−3^)	0.46, −0.28

Computer programs: *SMART* and *SAINT* (Bruker, 2012 ▸), *SHELXS* (Sheldrick, 2008 ▸), *SHELXL2014/7* (Sheldrick, 2015 ▸), *SHELXTL* (Sheldrick, 2008 ▸) and *publCIF* (Westrip, 2010 ▸).

## full crystallographic data

### Crystal data

**Table:**

C_22_H_22_N_2_O_4_	*F*(000) = 800
*M**_r_* = 378.42	*D*_x_ = 1.337 Mg m^−^^3^
Monoclinic, *P*2_1_/*n*	Mo *K*α radiation, λ = 0.71073 Å
Hall symbol: -P 2yn	Cell parameters from 2685 reflections
*a* = 9.6536 (9) Å	θ = 2.3–25.9°
*b* = 9.0435 (9) Å	µ = 0.09 mm^−^^1^
*c* = 21.599 (2) Å	*T* = 200 K
β = 94.473 (2)°	Block, colorless
*V* = 1879.9 (3) Å^3^	0.26 × 0.21 × 0.08 mm
*Z* = 4	

### Data collection

**Table:**

Bruker SMART CCD diffractometer	1933 reflections with *I* > 2σ(*I*)
Radiation source: fine-focus sealed tube	*R*_int_ = 0.055
Graphite monochromator	θ_max_ = 26.0°, θ_min_ = 1.9°
phi and ω scans	*h* = −11→11
11235 measured reflections	*k* = −11→10
3687 independent reflections	*l* = −21→26

### Refinement

**Table:**

Refinement on *F*^2^	Primary atom site location: structure-invariant direct methods
Least-squares matrix: full	Secondary atom site location: difference Fourier map
*R*[*F*^2^ > 2σ(*F*^2^)] = 0.059	Hydrogen site location: inferred from neighbouring sites
*wR*(*F*^2^) = 0.184	H-atom parameters constrained
*S* = 0.92	*w* = 1/[σ^2^(*F*_o_^2^) + (0.093*P*)^2^] where *P* = (*F*_o_^2^ + 2*F*_c_^2^)/3
3687 reflections	(Δ/σ)_max_ < 0.001
257 parameters	Δρ_max_ = 0.46 e Å^−^^3^
0 restraints	Δρ_min_ = −0.28 e Å^−^^3^

### Special details

**Table:**

Geometry. All esds (except the esd in the dihedral angle between two l.s. planes) are estimated using the full covariance matrix. The cell esds are taken into account individually in the estimation of esds in distances, angles and torsion angles; correlations between esds in cell parameters are only used when they are defined by crystal symmetry. An approximate (isotropic) treatment of cell esds is used for estimating esds involving l.s. planes.
Refinement. Refinement of F^2^ against ALL reflections. The weighted R-factor wR and goodness of fit S are based on F^2^, conventional R-factors R are based on F, with F set to zero for negative F^2^. The threshold expression of F^2^ > 2sigma(F^2^) is used only for calculating R-factors(gt) etc. and is not relevant to the choice of reflections for refinement. R-factors based on F^2^ are statistically about twice as large as those based on F, and R- factors based on ALL data will be even larger.

### Fractional atomic coordinates and isotropic or equivalent isotropic displacement parameters (Å^2^)

**Table:**

	*x*	*y*	*z*	*U*_iso_*/*U*_eq_	
O1	0.8751 (3)	1.3353 (3)	0.02640 (12)	0.0694 (7)	
H1	0.8035	1.2849	0.0310	0.104*	
C1	0.9888 (3)	1.2574 (4)	0.04692 (14)	0.0506 (8)	
C2	0.9795 (3)	1.1101 (3)	0.06640 (12)	0.0398 (7)	
C3	1.1048 (3)	1.0390 (4)	0.08642 (13)	0.0501 (8)	
C4	1.2308 (3)	1.1127 (5)	0.08911 (15)	0.0671 (11)	
H4	1.3138	1.0633	0.1038	0.080*	
C5	1.2350 (4)	1.2572 (6)	0.07051 (17)	0.0747 (13)	
H5	1.3216	1.3076	0.0726	0.090*	
C6	1.1167 (4)	1.3309 (4)	0.04891 (17)	0.0701 (11)	
H6	1.1215	1.4306	0.0354	0.084*	
O2	1.0935 (2)	0.8949 (3)	0.10310 (11)	0.0653 (7)	
C7	1.2171 (3)	0.8106 (5)	0.11670 (17)	0.0791 (13)	
H7A	1.2748	0.8154	0.0814	0.119*	
H7B	1.1924	0.7074	0.1243	0.119*	
H7C	1.2689	0.8511	0.1537	0.119*	
C8	0.8433 (3)	1.0377 (3)	0.06725 (11)	0.0335 (6)	
C9	0.8141 (3)	0.8850 (3)	0.09064 (13)	0.0373 (7)	
H9A	0.8446	0.8750	0.1353	0.045*	
H9B	0.8610	0.8086	0.0669	0.045*	
C10	0.6550 (3)	0.8726 (3)	0.07970 (12)	0.0345 (7)	
H10	0.6331	0.8009	0.0451	0.041*	
N1	0.7304 (2)	1.1080 (3)	0.04944 (10)	0.0396 (6)	
N2	0.6126 (2)	1.0220 (3)	0.05752 (11)	0.0441 (6)	
H2A	0.5262	1.0522	0.0505	0.053*	
C11	0.5866 (2)	0.8181 (3)	0.13617 (11)	0.0320 (6)	
C12	0.5293 (3)	0.6788 (3)	0.13404 (12)	0.0364 (7)	
C13	0.4716 (3)	0.6134 (3)	0.18643 (13)	0.0405 (7)	
C14	0.4723 (3)	0.6915 (3)	0.23996 (13)	0.0442 (8)	
H14	0.4354	0.6478	0.2751	0.053*	
C15	0.5264 (3)	0.8358 (3)	0.24460 (12)	0.0401 (7)	
C16	0.5249 (3)	0.9182 (4)	0.30062 (14)	0.0544 (9)	
H16	0.4853	0.8758	0.3354	0.065*	
C17	0.5787 (3)	1.0556 (4)	0.30515 (15)	0.0596 (9)	
H17	0.5734	1.1103	0.3424	0.072*	
C18	0.6427 (3)	1.1186 (4)	0.25521 (15)	0.0561 (9)	
H18	0.6841	1.2137	0.2595	0.067*	
C19	0.6453 (3)	1.0430 (3)	0.20025 (14)	0.0473 (8)	
H19	0.6875	1.0876	0.1666	0.057*	
C20	0.5872 (3)	0.9015 (3)	0.19266 (12)	0.0361 (7)	
O3	0.51575 (19)	0.5996 (2)	0.07876 (9)	0.0458 (6)	
C21	0.6160 (4)	0.4843 (4)	0.07440 (16)	0.0641 (10)	
H21A	0.7097	0.5268	0.0779	0.096*	
H21B	0.5998	0.4341	0.0343	0.096*	
H21C	0.6073	0.4129	0.1080	0.096*	
O4	0.4194 (2)	0.4742 (2)	0.17676 (10)	0.0552 (6)	
C22	0.3611 (4)	0.4043 (4)	0.22780 (17)	0.0702 (11)	
H22A	0.4324	0.3948	0.2624	0.105*	
H22B	0.3269	0.3059	0.2152	0.105*	
H22C	0.2839	0.4641	0.2409	0.105*	

### Atomic displacement parameters (Å^2^)

**Table:**

	*U*^11^	*U*^22^	*U*^33^	*U*^12^	*U*^13^	*U*^23^
O1	0.0720 (17)	0.0475 (15)	0.0932 (19)	−0.0040 (13)	0.0345 (15)	0.0078 (13)
C1	0.053 (2)	0.054 (2)	0.0474 (19)	−0.0117 (17)	0.0242 (15)	−0.0107 (16)
C2	0.0361 (16)	0.055 (2)	0.0295 (15)	−0.0084 (15)	0.0084 (12)	−0.0068 (14)
C3	0.0368 (18)	0.079 (3)	0.0350 (17)	−0.0054 (17)	0.0065 (13)	−0.0057 (16)
C4	0.0354 (19)	0.121 (4)	0.045 (2)	−0.014 (2)	0.0082 (15)	−0.011 (2)
C5	0.054 (2)	0.120 (4)	0.053 (2)	−0.042 (3)	0.0256 (18)	−0.036 (2)
C6	0.079 (3)	0.072 (3)	0.064 (2)	−0.034 (2)	0.037 (2)	−0.024 (2)
O2	0.0289 (12)	0.096 (2)	0.0708 (16)	0.0136 (12)	0.0008 (10)	0.0202 (14)
C7	0.0374 (19)	0.132 (4)	0.067 (2)	0.027 (2)	−0.0023 (17)	0.014 (2)
C8	0.0325 (15)	0.0428 (17)	0.0259 (14)	−0.0002 (13)	0.0057 (11)	−0.0012 (12)
C9	0.0307 (15)	0.0443 (17)	0.0373 (16)	0.0040 (13)	0.0057 (12)	0.0031 (13)
C10	0.0331 (15)	0.0417 (17)	0.0288 (15)	−0.0007 (13)	0.0035 (11)	−0.0003 (13)
N1	0.0344 (13)	0.0459 (15)	0.0393 (14)	0.0032 (12)	0.0074 (10)	0.0079 (11)
N2	0.0241 (12)	0.0538 (16)	0.0536 (16)	0.0054 (11)	−0.0020 (10)	0.0145 (12)
C11	0.0213 (13)	0.0447 (17)	0.0297 (15)	0.0025 (12)	0.0001 (11)	−0.0004 (12)
C12	0.0302 (15)	0.0444 (18)	0.0349 (16)	0.0035 (13)	0.0050 (12)	−0.0011 (13)
C13	0.0355 (16)	0.0420 (18)	0.0438 (18)	0.0007 (14)	0.0028 (13)	0.0044 (14)
C14	0.0360 (16)	0.059 (2)	0.0376 (18)	0.0008 (15)	0.0018 (13)	0.0097 (15)
C15	0.0308 (15)	0.061 (2)	0.0281 (15)	0.0051 (14)	−0.0006 (12)	−0.0056 (14)
C16	0.0481 (19)	0.077 (3)	0.0387 (18)	−0.0045 (18)	0.0085 (14)	−0.0060 (17)
C17	0.063 (2)	0.074 (3)	0.043 (2)	−0.004 (2)	0.0132 (17)	−0.0211 (18)
C18	0.0483 (19)	0.059 (2)	0.060 (2)	−0.0057 (16)	0.0004 (16)	−0.0177 (18)
C19	0.0406 (17)	0.059 (2)	0.0434 (18)	−0.0050 (16)	0.0088 (14)	−0.0124 (16)
C20	0.0263 (14)	0.0462 (18)	0.0360 (16)	−0.0035 (13)	0.0034 (12)	−0.0051 (13)
O3	0.0458 (12)	0.0512 (13)	0.0400 (12)	−0.0005 (10)	0.0016 (9)	−0.0119 (10)
C21	0.065 (2)	0.064 (2)	0.063 (2)	0.0168 (19)	0.0026 (18)	−0.0185 (18)
O4	0.0632 (14)	0.0494 (14)	0.0546 (14)	−0.0158 (12)	0.0142 (11)	0.0026 (11)
C22	0.073 (2)	0.062 (2)	0.077 (3)	−0.016 (2)	0.016 (2)	0.016 (2)

### Geometric parameters (Å, º)

**Table:**

O1—C1	1.350 (4)	C11—C12	1.376 (4)
O1—H1	0.8400	C11—C20	1.434 (4)
C1—C6	1.400 (5)	C12—O3	1.390 (3)
C1—C2	1.402 (4)	C12—C13	1.428 (4)
C2—C3	1.407 (4)	C13—C14	1.354 (4)
C2—C8	1.470 (4)	C13—O4	1.365 (3)
C3—O2	1.358 (4)	C14—C15	1.406 (4)
C3—C4	1.385 (4)	C14—H14	0.9500
C4—C5	1.369 (5)	C15—C16	1.422 (4)
C4—H4	0.9500	C15—C20	1.435 (4)
C5—C6	1.372 (5)	C16—C17	1.347 (4)
C5—H5	0.9500	C16—H16	0.9500
C6—H6	0.9500	C17—C18	1.405 (5)
O2—C7	1.427 (4)	C17—H17	0.9500
C7—H7A	0.9800	C18—C19	1.372 (4)
C7—H7B	0.9800	C18—H18	0.9500
C7—H7C	0.9800	C19—C20	1.402 (4)
C8—N1	1.295 (3)	C19—H19	0.9500
C8—C9	1.505 (4)	O3—C21	1.431 (3)
C9—C10	1.539 (4)	C21—H21A	0.9800
C9—H9A	0.9900	C21—H21B	0.9800
C9—H9B	0.9900	C21—H21C	0.9800
C10—N2	1.481 (3)	O4—C22	1.424 (4)
C10—C11	1.514 (4)	C22—H22A	0.9800
C10—H10	1.0000	C22—H22B	0.9800
N1—N2	1.400 (3)	C22—H22C	0.9800
N2—H2A	0.8800		
			
C1—O1—H1	109.5	C12—C11—C20	119.0 (2)
O1—C1—C6	117.1 (3)	C12—C11—C10	118.1 (2)
O1—C1—C2	121.7 (3)	C20—C11—C10	122.8 (2)
C6—C1—C2	121.2 (3)	C11—C12—O3	120.7 (2)
C1—C2—C3	116.9 (3)	C11—C12—C13	122.3 (3)
C1—C2—C8	120.3 (3)	O3—C12—C13	116.8 (2)
C3—C2—C8	122.7 (3)	C14—C13—O4	126.1 (3)
O2—C3—C4	122.6 (3)	C14—C13—C12	118.9 (3)
O2—C3—C2	115.8 (3)	O4—C13—C12	115.0 (3)
C4—C3—C2	121.6 (4)	C13—C14—C15	121.5 (3)
C5—C4—C3	119.6 (4)	C13—C14—H14	119.3
C5—C4—H4	120.2	C15—C14—H14	119.3
C3—C4—H4	120.2	C14—C15—C16	121.2 (3)
C4—C5—C6	121.4 (3)	C14—C15—C20	120.1 (3)
C4—C5—H5	119.3	C16—C15—C20	118.7 (3)
C6—C5—H5	119.3	C17—C16—C15	121.0 (3)
C5—C6—C1	119.3 (4)	C17—C16—H16	119.5
C5—C6—H6	120.4	C15—C16—H16	119.5
C1—C6—H6	120.4	C16—C17—C18	120.6 (3)
C3—O2—C7	119.0 (3)	C16—C17—H17	119.7
O2—C7—H7A	109.5	C18—C17—H17	119.7
O2—C7—H7B	109.5	C19—C18—C17	120.1 (3)
H7A—C7—H7B	109.5	C19—C18—H18	120.0
O2—C7—H7C	109.5	C17—C18—H18	120.0
H7A—C7—H7C	109.5	C18—C19—C20	121.4 (3)
H7B—C7—H7C	109.5	C18—C19—H19	119.3
N1—C8—C2	120.7 (3)	C20—C19—H19	119.3
N1—C8—C9	112.0 (2)	C19—C20—C11	123.7 (3)
C2—C8—C9	127.2 (2)	C19—C20—C15	118.1 (3)
C8—C9—C10	103.1 (2)	C11—C20—C15	118.2 (3)
C8—C9—H9A	111.1	C12—O3—C21	114.4 (2)
C10—C9—H9A	111.1	O3—C21—H21A	109.5
C8—C9—H9B	111.1	O3—C21—H21B	109.5
C10—C9—H9B	111.1	H21A—C21—H21B	109.5
H9A—C9—H9B	109.1	O3—C21—H21C	109.5
N2—C10—C11	115.5 (2)	H21A—C21—H21C	109.5
N2—C10—C9	103.3 (2)	H21B—C21—H21C	109.5
C11—C10—C9	113.1 (2)	C13—O4—C22	117.0 (3)
N2—C10—H10	108.2	O4—C22—H22A	109.5
C11—C10—H10	108.2	O4—C22—H22B	109.5
C9—C10—H10	108.2	H22A—C22—H22B	109.5
C8—N1—N2	111.3 (2)	O4—C22—H22C	109.5
N1—N2—C10	109.9 (2)	H22A—C22—H22C	109.5
N1—N2—H2A	125.0	H22B—C22—H22C	109.5
C10—N2—H2A	125.0		
			
O1—C1—C2—C3	179.5 (3)	C9—C10—C11—C20	65.6 (3)
C6—C1—C2—C3	−1.6 (4)	C20—C11—C12—O3	174.4 (2)
O1—C1—C2—C8	−2.4 (4)	C10—C11—C12—O3	−9.6 (4)
C6—C1—C2—C8	176.5 (3)	C20—C11—C12—C13	−0.7 (4)
C1—C2—C3—O2	−177.9 (2)	C10—C11—C12—C13	175.3 (2)
C8—C2—C3—O2	4.1 (4)	C11—C12—C13—C14	0.5 (4)
C1—C2—C3—C4	2.5 (4)	O3—C12—C13—C14	−174.8 (2)
C8—C2—C3—C4	−175.6 (3)	C11—C12—C13—O4	−179.7 (2)
O2—C3—C4—C5	178.8 (3)	O3—C12—C13—O4	5.0 (3)
C2—C3—C4—C5	−1.6 (5)	O4—C13—C14—C15	−178.7 (3)
C3—C4—C5—C6	−0.3 (5)	C12—C13—C14—C15	1.0 (4)
C4—C5—C6—C1	1.2 (5)	C13—C14—C15—C16	179.0 (3)
O1—C1—C6—C5	178.8 (3)	C13—C14—C15—C20	−2.3 (4)
C2—C1—C6—C5	−0.2 (5)	C14—C15—C16—C17	179.0 (3)
C4—C3—O2—C7	−7.9 (4)	C20—C15—C16—C17	0.3 (4)
C2—C3—O2—C7	172.5 (3)	C15—C16—C17—C18	−2.5 (5)
C1—C2—C8—N1	0.8 (4)	C16—C17—C18—C19	2.9 (5)
C3—C2—C8—N1	178.8 (3)	C17—C18—C19—C20	−1.1 (5)
C1—C2—C8—C9	−175.6 (3)	C18—C19—C20—C11	179.7 (3)
C3—C2—C8—C9	2.4 (4)	C18—C19—C20—C15	−1.1 (4)
N1—C8—C9—C10	3.2 (3)	C12—C11—C20—C19	178.7 (2)
C2—C8—C9—C10	179.8 (2)	C10—C11—C20—C19	2.8 (4)
C8—C9—C10—N2	−5.2 (3)	C12—C11—C20—C15	−0.5 (4)
C8—C9—C10—C11	−130.8 (2)	C10—C11—C20—C15	−176.4 (2)
C2—C8—N1—N2	−176.4 (2)	C14—C15—C20—C19	−177.3 (2)
C9—C8—N1—N2	0.5 (3)	C16—C15—C20—C19	1.5 (4)
C8—N1—N2—C10	−4.2 (3)	C14—C15—C20—C11	2.0 (4)
C11—C10—N2—N1	129.9 (2)	C16—C15—C20—C11	−179.2 (2)
C9—C10—N2—N1	5.8 (3)	C11—C12—O3—C21	103.7 (3)
N2—C10—C11—C12	130.9 (3)	C13—C12—O3—C21	−80.9 (3)
C9—C10—C11—C12	−110.3 (3)	C14—C13—O4—C22	−0.4 (4)
N2—C10—C11—C20	−53.2 (3)	C12—C13—O4—C22	179.8 (3)

### Hydrogen-bond geometry (Å, º)

**Table:**

*D*—H···*A*	*D*—H	H···*A*	*D*···*A*	*D*—H···*A*
O1—H1···N1	0.84	1.81	2.556 (3)	148
N2—H2*A*···N2^i^	0.88	2.68	3.196 (5)	118
C6—H6···O1^ii^	0.95	2.50	3.433 (5)	166

Symmetry codes: (i) −*x*+1, −*y*+2, −*z*; (ii) −*x*+2, −*y*+3, −*z*.
